# Reduction of formaldehyde emission from urea-formaldehyde resin with a small quantity of graphene oxide

**DOI:** 10.1039/d1ra06717f

**Published:** 2021-10-05

**Authors:** Kazuki Saito, Yasushi Hirabayashi, Shinya Yamanaka

**Affiliations:** Division of Applied Sciences, Muroran Institute of Technology Mizumoto-cho 27-1 Muroran 050-8585 Japan syama@mmm.muroran-it.ac.jp; Forest Products Research Institute, Hokkaido Research Organization Nishikagura 1-10 Asahikawa 071-0198 Japan

## Abstract

Graphene oxide (GO) has theoretically been identified as a candidate for adsorbing formaldehyde molecules. However, whether GO can actually serve as a scavenger for formaldehyde resin adhesives must be experimentally verified due to the complex interaction between GO and formaldehyde molecules in the presence of resin, the competition between the formaldehyde emission rate and its adsorption rate on the scavenger, and other complications. From the results from this study we experimentally demonstrate that GO synthesised by the improved Hummers' method is a powerful scavenger for a urea–formaldehyde (UF) resin. We investigate the effect of the added amount of GO on the formaldehyde emission from UF resin. The emission from the UF/GO composite resin is 0.22 ± 0.03 mg L^−1^, which is an 81.5% reduction compared to that of the control UF resin when adding 0.20 wt% GO into the UF resin. However, adding higher amounts of GO (more than 0.20 wt%) increases the formaldehyde emission and the emission approaches that of pure UF resin (1.19 ± 0.36 mg L^−1^). This is likely due to the more acidic pH of the composite, which may lead to a faster curing reaction of the UF resin and acceleration of the emission.

## Abstract 2

This is the first experiment to demonstrate that GO effectively prevents formaldehyde emission from UF resin.

## Graphical abstract

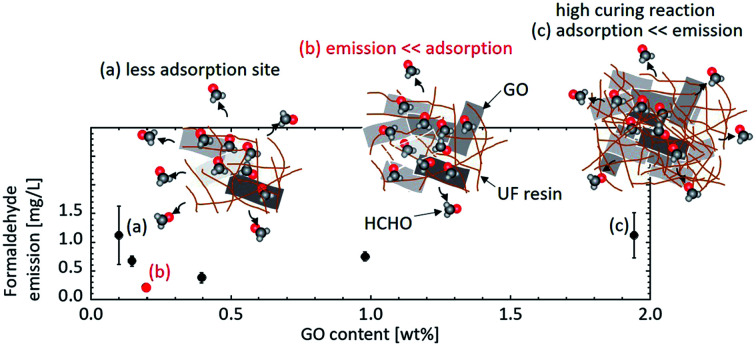

## Introduction

All formaldehyde-based adhesives emit formaldehyde, which is a serious drawback. In 2004, such additives were reclassified as a Group 1 human carcinogen by the International Agency for Research on Cancer. Consequently, formaldehyde is regulated in indoor environments.^1^ Several plywood adhesives emit formaldehyde due to hydrolysis of weak chemical bonds both during the production and long-term use of wood-based materials. To date, governments in Europe,^2^ Australia and New Zealand,^3,4^ the United States,^5,6^ and Japan^7,8^ have implemented standards to regulate formaldehyde emission.

Urea–formaldehyde (UF) resin is a practical adhesive used to manufacture wood-based panels such as particleboard, fibreboard, and plywood.^9^ Its advantages include economical viability, fast reaction time in a hot press, water solubility, low curing temperature, resistance to microorganisms and to abrasion, and its colourless, especially cured resins.^10^ Although UF resins are extensively applied as bonding agents in diverse applications, reduction of formaldehyde emission from wood-based panels in the environment remains a critical challenge in the industry.^11^

One promising technique to reduce the formaldehyde emission from wood-based panels is to add scavengers (catchers) such as natural compounds or amine compounds.^12–14^ Among these scavengers, urea is highly reactive and rapidly forms a strong bond. Adsorption is another effective method to inhibit formaldehyde emission. The addition of urea, other compounds like ammonium salts^15^ and inorganic nanoparticles^16,17^ can also be used. P. H. G. de Cademartori *et al.* investigated the addition of alumina nanoparticles into UF resin.^16^ They concluded that the nanoparticles reduced formaldehyde emission during UF curing and at environmental temperatures. The effect of TiO_2_ nanoparticles loading on formaldehyde emission were investigated by Y. Liu and X. Zhu.^17^ The use of natural, bio-based scavengers such as tannins,^18,19^ hydrolysis lignin,^20^ ammonium lignosulfonate,^21^ and cellulose^22^ has been studied to not only reduce formaldehyde emission but also improve the adhesion properties. These scavengers were summarized by review of the literatures.^10,23^

Most studies in the literature focus on the adsorption of formaldehyde on activated carbons^24–29^ and other carbon-based nanomaterials.^30–32^ Especially carbon-based adsorbents modified by various groups have been widely studied, and currently appear to be the most effective and practical way to remove formaldehyde.^33^

The physical and mechanical characteristics of wood-based panels reinforced with the addition of inorganic nanoparticles^34,35^ and carbon-based materials.^30–32^ A. Kumar *et al.* reported the effects of activated charcoal^30^ and multi-walled carbon nanotubes^31,32^ on the physical and mechanical properties of a medium density fiberboard. These carbon-based materials had an accelerating effect on the curing of the UF resin.

Graphene oxide (GO) is usually obtained through the oxidation of graphite by the Hummers' method.^36,37^ Compared with other carbon-based materials, GO has a high specific surface area and a folded structure.^38,39^ Thus, it can provide a huge capacity for absorbing pollutants. Density functional theory (DFT) studies are performed to understand the adsorption property of the pollutant molecules on different materials at the electronic, atomic, and molecular levels. In the past decade, many theoretical studies have addressed formaldehyde on carbon-based materials,^40–46^ including the interaction of formaldehyde with GO.^41^ Although these DFT studies have noted that GO has an excellent adsorption capacity for formaldehyde, GO adsorption on formaldehyde-resin has yet to be experimentally investigated. Very recently, W. Gul and H. Alrobei reported the physical and mechanical properties of medium density fiberboard enhanced with graphene oxide.^47^ However, they did not measure the formaldehyde emission.

Lee and colleague have suggested that surface functional groups, including oxygen atoms, of activated carbon fibres decrease the adsorbed amount of formaldehyde in humid conditions due to their affinity to water.^48^ Thus, an intermediary resin and a formaldehyde emission kinetics during the hydrolysis reaction of resin make it difficult to reproduce the DFT predictions because DFT studies focus on an ideal system (*i.e.*, the interaction between the formaldehyde molecule and functional groups on the GO surface).

Herein we demonstrate that GO is an excellent scavenger and propose a new composite UF-based adhesive. The proposed UF/GO resin exhibits a low formaldehyde emission.

We not only investigate the effect of GO addition on the formaldehyde emission from UF resins, but also show that the pH of UF/GO resins plays a crucial role in extracting the GO ability.

## Results and discussion

### Characterisation of GO

**Fig. 1:**
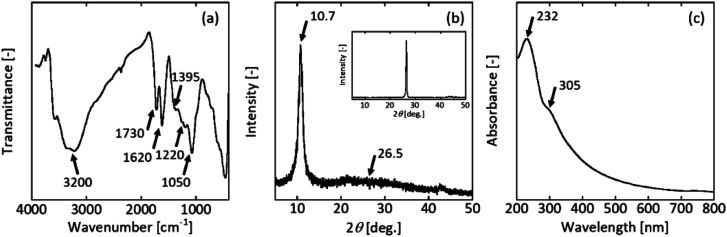
(a) FT-IR spectrum, (b) XRD pattern, and (c) UV-vis spectrum of GO prepared by the improved Hummers' method. The insert figure in (b) is XRD pattern of raw graphite.

The GO sample contained oxygen functional groups. The FT-IR spectrum exhibited a broad peak and high-frequency area around 3200 cm^−1^, which were both attributed to the O–H stretching mode, indicating the presence of a hydroxyl group on GO (Fig. 1(a)). The bands at wavenumbers of 1730 cm^−1^ and 1620 cm^−1^ corresponded to the C=O stretching (carbonyl group) and C=C stretching vibration, respectively. The peaks at 1395 cm^−1^, 1220 cm^−1^, and 1050 cm^−1^ represented the C–OH, C–O–C, and C–O stretching frequencies, respectively. These peaks agreed well with the literature.^49,50^

Graphite as the raw material had a sharp diffraction peak at 2*θ* = 26.5°. This peak corresponded to the (002) plane of hexagonal graphite structure. XRD analysis confirmed the crystalline nature and phase purity of the synthesised GO (Fig. 1(b)). The relatively wide diffraction peak at 10.7° corresponded to GO,^49,50^ revealing an expansion of the interlayer spacing from 0.34 nm (graphite) to ∼0.8 nm. The peak at 26.5° disappeared, confirming that almost all the raw graphite was converted to GO.

The UV-vis spectrum had a strong absorption peak at 232 nm (Fig. 1(c)). This peak was attributed to the π–π* transition of the C–C conjugated aromatic domains and weak absorption with a shoulder at 305 nm due to n–π* transition of C=O bond. The UV-vis spectrum with GO peaks at 232 nm underwent a colour change from black to brown.^50^

**Fig. 2:**
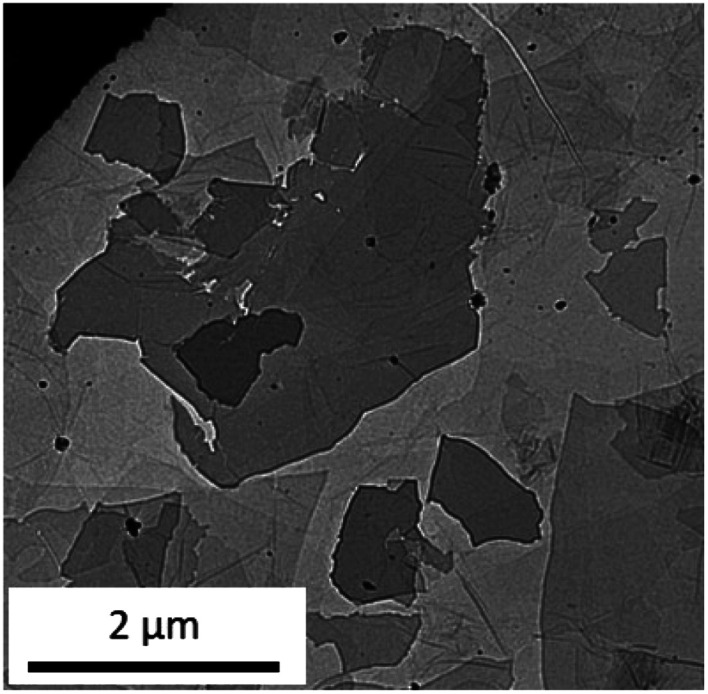
Typical TEM image of the prepared GO.

Fig. 2 depicts a typical TEM image of GO. Submicron to several microns of a few layer sheets were observed. The FT-IR, XRD, UV-vis, and TEM observations all provided evidence that the prepared sample contained a few layers of GO.

### Formaldehyde emission from UF/GO resin

The formaldehyde emission was 1.19 ± 0.36 mg L^−1^ for the UF resin only. The addition of graphite with 0.20–1.9 wt% to the total weight of the solid UF resin, graphite, and curing agent had an emission of 1.27 ± 0.43 mg L^−1^. The emission did not significantly change because the loading amount of raw graphite was too small to adsorb formaldehyde. Previously we investigated the reduction of the formaldehyde emission from a UF/natural scavenger (scallop shell nanoparticles, main component was calcium carbonate).^51^ To reduce the emission, the contained particles should be over 80 wt% of the total weight of the solid content of UF resin and the scavenger (Table 1). Herein a graphite amount of 1.9 wt% or less was insufficient to prevent formaldehyde emission.

**Table 1:** Comparison of formaldehyde emission from UF/scavenger resins

Scavengers	Added amount [wt%]	Formaldehyde emission [mg L^−1^]		Rate of decrease [%]	Ref.
		With scavengers^d^	Without scavenger		
Urea modified scallop shell	83.8^a^	3.9	11.4	65.8	51
Propylamine	0.7^a^	0.32	0.7	54.3	53
Chitosan nanoparticles	1^a^	0.22	0.54	59.3	54
Alumina nanoparticles	2^a^	3.7 ppm	4.3 ppm	14.0	16
Copolymer	7.5^b^	1.20	2.00	40.0	55
Pozzolan	10^b^	5.3	9.9	46.5	56
Ethyl cellulose microcapsules	68.1^b^	0.49	1.37	64.2	57
Multiwalled carbon nanotubes	0.52^c^	7.7^e^	12.3^e^	37.4	30
GO	0.20^a^	0.22	1.19	81.5	This study

a Based on the total weight of the solid resin, the scavenger, and the curing agent.

b Unknown whether based on (a) or not.

c Volume percent.

d It is noted that the curing reaction was carried out using an oven in this study. According to our previous study,^58^ this simple evaluation method for measuring a formaldehyde emission from UF resin was in good agreement with the emission from plywood.

e The unit is mg/100 g.

**Fig. 3:**
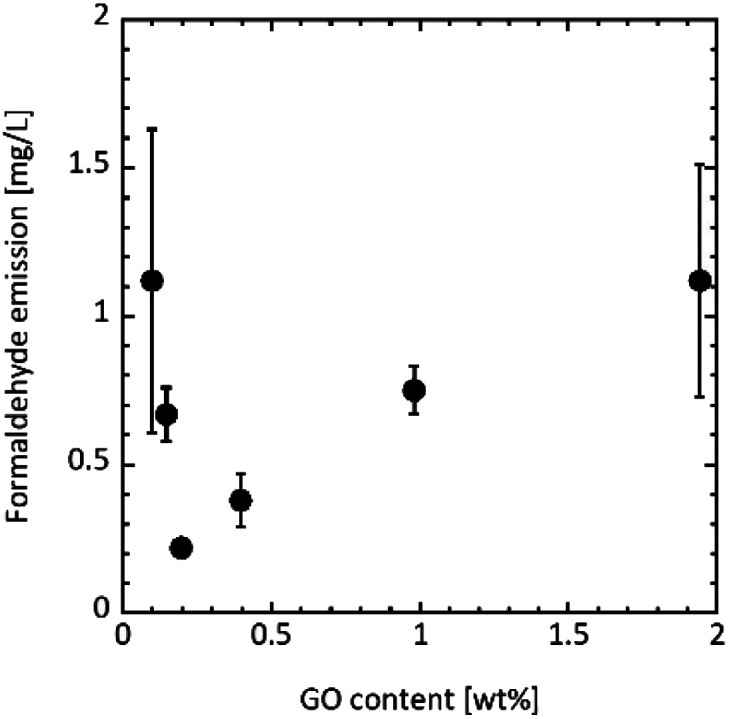
Formaldehyde emission from UF/GO resins measured according to the desiccator method^47^ for different GO contents.

Fig. 3 shows the formaldehyde emission from UF/GO. Formaldehyde emissions of 1.12 ± 0.51, 0.67 ± 0.09, and 0.22 ± 0.03 mg L^−1^ were achieved upon adding 0.10, 0.15, and 0.20 wt% of GO, respectively. The emission clearly decreased compared with the UF and UF/graphite resin. In particular, 0.20 wt% GO addition gave the lowest formaldehyde emission of 0.22 ± 0.03 mg L^−1^.

It should be noted that the –OH and –COOH groups of GO may react with free formaldehyde in the resins. According to DFT calculations, M. D. Esrafili and L. Dinparast has been pointed out the most stable adsorption configuration of formaldehyde is when it interacts with O atoms of surface *via* its H atom.^41^ Additionally, its adsorption energy is very low (−2.1 kcal mol^−1^) indicating the interaction of formaldehyde molecule with the GO surface is physisorption.^41^ On the other hand, M. Chavali *et al.* performed molecular dynamics simulation for formaldehyde–graphene oxide system.^52^ They reported that one formaldehyde molecule of adsorption heat was −76.4 kcal mol^−1^, which was close to the chemical adsorption.

Although an adsorption mechanism of formaldehyde on GO surface is not clear, we are not convinced that small quantity of GO could reduce more than 80% formaldehyde emission since there is physical adsorption between them only. Formaldehyde may react with the –OH and –COOH groups on the GO surface.

Previous studies have employed various materials, including natural, carbon-based, and other inorganic/organic materials, as a scavenger. Table 1 lists formaldehyde emission from the UF/scavenger resins. Here, even a small amount of added GO produced a high reduction effect. It should be noted that the reported formaldehyde emission from the control UF resin varies in the literature (without scavenger in Table 1). In this study, the resin with a 0.20 wt% GO content had an average formaldehyde emission of 0.22 mg L^−1^, which was an 81.5% reduction compared to that of the control UF resin. Additionally, the decrease ratio of the formaldehyde emission was quite high compared with previously reported scavengers.

DFT calculations have predicted that GO is a potential candidate for excellent formaldehyde adsorbent.^41^ However, this is the first experiment to demonstrate that GO effectively prevents formaldehyde emission from UF resin.

Increasing the GO content did not decrease the formaldehyde emission. The emission was 0.38 ± 0.09, 0.75 ± 0.08, and 1.12 ± 0.39 mg L^−1^ for a GO content of 0.40, 1.0, and 1.9 wt%, respectively. Moreover, formaldehyde was emitted at almost the same level as the UF resin (1.19 ± 0.36 mg L^−1^) with a GO amount of 1.9 wt%. Xing *et al.* have reported the effect of pH value on the UF resin gel time. They demonstrated that the gel time of the UF resin exponentially decreased with decreasing pH.^59^Table 3 lists the pH values for each UF/GO liquid after the curing treatment. The pH gradually decreased as the GO addition amount increased, indicating that the UF resin before curing was more acidic. It is thought that the rate of formaldehyde emission is accelerated in acidic conditions due to the faster curing reaction.

**Fig. 4:**
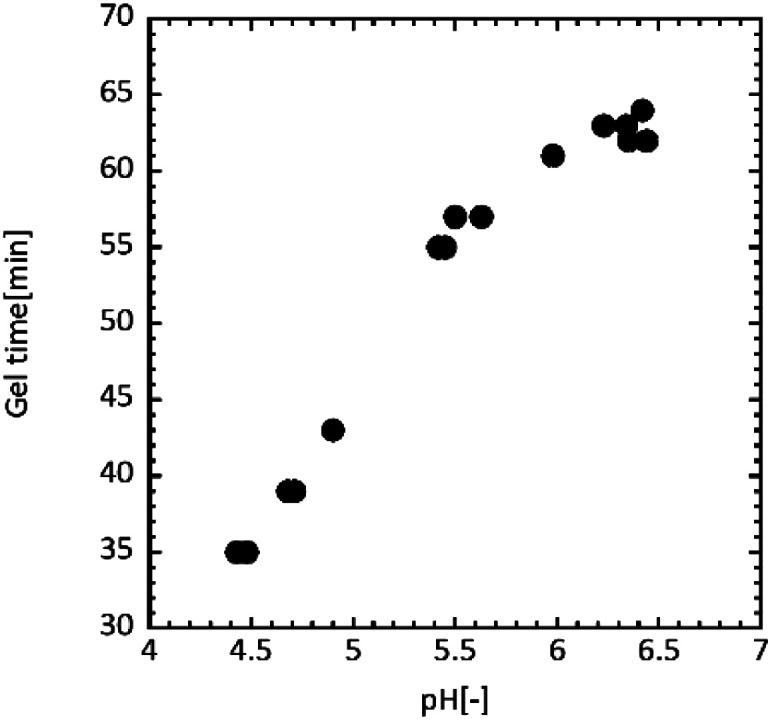
pH dependency of UF resin gel time.

Fig. 4 shows the relation between pH and the gel time for UF resin. When the pH was adjusted around 4.5, the gel time was 35–40 min, while the gel time was 60–65 min at pH = 6.0–6.5. The faster curing was observed under acidic conditions, indicating an acceleration of formaldehyde emission.

With 0.20, and 1.9 wt% GO content, the pH of UF/GO was regulated (see Experimental section: preparation of urea resin/GO and test for formaldehyde emission). As shown in Table 2, the formaldehyde emission dramatically increased from 0.22 ± 0.03 mg L^−1^ (unadjusted pH) to 0.91 ± 0.09 mg L^−1^ (adjusted pH) with 0.20 wt% GO content. On the other hand, the emission dramatically decreased from 1.12 ± 0.39 mg L^−1^ (unadjusted pH) to 0.43 ± 0.05 mg L^−1^ (adjusted pH) with 1.9 wt% GO content. When the pH of UF/GO was adjusted, the formaldehyde emission was improved or worsened, suggesting that the formaldehyde emission is sensitive to the pH of the UF/GO resin.

**Table 2:** Formaldehyde emission with and without pH regulation

GO content [wt%]	Formaldehyde emissions [mg L^−1^]	
	pH unadjusted	pH adjusted
0.20	0.22 ± 0.03	0.91 ± 0.09^a^
1.9	1.12 ± 0.39	0.43 ± 0.05^b^

a pH was adjusted to 4.73, which was the same as that of the GO content 1.9 wt%.

b pH was adjusted to the same as that of the GO content 0.20 wt%.

**Fig. 5:**
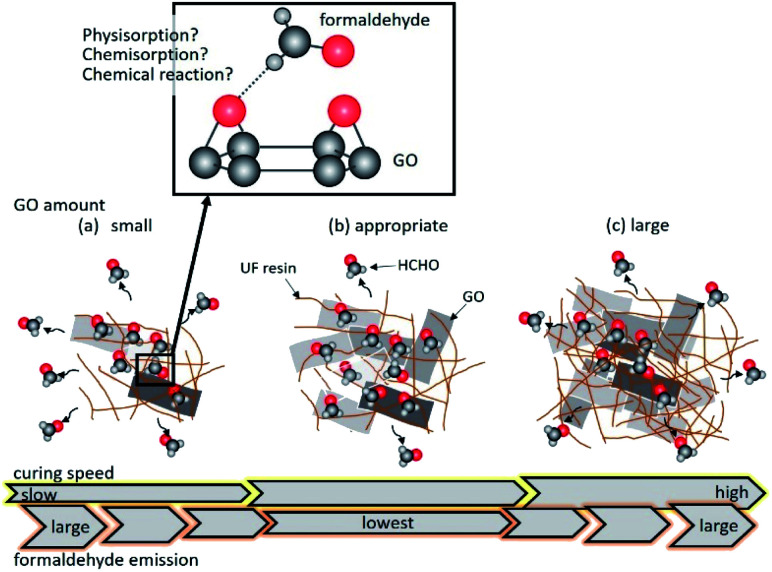
Illustration of the formaldehyde emission from UF/GO resins containing (a) 0.10, 0.15 wt%, (b) 0.20 wt%, and (c) 0.40, 1.0, 1.9 wt% GO. The upper magnified image expresses an interaction of O atom of GO surface with H atom of formaldehyde based on the knowledge of ref. 41 and 52.

Fig. 5 illustrates the effect of GO addition on the formaldehyde emission from the UF resin. Similar to the case of adding raw graphite, formaldehyde adsorption did not proceed when the amount of GO was small due to the limited number of GO adsorption sites (Fig. 5(a)). However, in the case of adequate GO addition into the UF resin, GO could adsorb formaldehyde before it diffused (Fig. 5(b)). The pH of urea resin dropped to the acidic conditions when the amount of GO was large. It is speculated that the curing is more likely to occur and formaldehyde more predominantly diffuses into the atmosphere than is adsorbed on the GO surface (Fig. 5(c)).

Therefore, even if a large amount of GO is added, formaldehyde emission is not reduced.

This study suggests that the pH of UF/GO must be carefully regulated. Although we should investigate adhesive strength of plywood, and also formaldehyde emission from plywood using the UF/GO resin, GO will be a potential candidate for scavenger of plywood production. We believe that this study opens a new practical application of GO as an adhesive scavenger.

## Experimental

### Materials

Natural graphite powder (CFW-18AK, 18 μm nominal particle size), and UF resin were provided by Chuetsu Graphite Works (Osaka, Japan), and DIC Kitanihon Polymer (Tomakomai, Japan), respectively. According to the manufacturer, the resin has the following general physicochemical properties: 51% non-volatile solids content, 7.5 pH, 110 mPa s viscosity, and 1.2 formaldehyde/urea molar ratio. Ammonium chloride (99.5% purity), which is a curing agent, was purchased from Kanto Chemical (Tokyo, Japan). Citric acid (>99.5% purity), and calcium hydroxide (>96.0% purity) as a pH adjuster were purchased from Wako Pure Chemical Industries (Osaka, Japan), and Kanto Chemical, respectively. Sulfuric acid (96.0% concentration), potassium permanganate (99.3% purity), and hydrogen peroxide solution (34.5% concentration) for GO preparation were purchased from Kanto Chemical. All reagents were used as received without further purification.

### Preparation of GO

GO was prepared from natural graphite powder *via* the improved Hummers' method.^37^ Briefly, 3 g of natural graphite powder was soaked in 276 g of sulfuric acid and subsequently reacted with 9 g of potassium permanganate in an ice bath for 2 h. The obtained solution was put into a mixture composed of 100 mL ion-exchanged water and 5 mL hydrogen peroxide solution to terminate the reaction.

The obtained suspension was centrifuged at 9280 × *g* and washed with ion-exchanged water. This operation was repeated at least three times. After the final centrifugation, the supernatant was discarded and the wet GO sediment was processed into powder by freeze-drying.

### Characterisation of prepared GO

The specific surface area of the resultant GO sample was determined by nitrogen gas adsorption based on the multi-point BET method. The analysis was conducted on Autosorb-1-c/MK2 (Qantachrome, USA). Prior to the measurement, the sample was degassed for 2 h at 200 °C under a vacuum to remove adsorbed solvent molecules. As the result the specific surface area of the resultant GO was 9.9 m^2^ g^−1^.

To study the crystal phase, functional groups, transparency, and exfoliation level, the sample was characterised by XRD, FT-IR, UV-vis, and TEM, respectively.

To estimate the surface functional group on the GO powder, the FTIR spectra (FT/IR-460PlusK; JASCO, Tokyo, Japan) were acquired using a KBr pellet technique with a scan range from 400 to 4000 cm^−1^. The KBr pellets contained 1–2 wt% of the GO powder. X-ray diffractometer, XRD (MultiFlex; Rigaku, Tokyo, Japan) powder pattern of the GO powder was obtained with Cu Kα radiation (40 kV, 20 mA). The scanning rate was set at 5° min^−1^ from 5° to 50°. The GO powder was placed on a reflection-free sample holder. For the diluted GO dispersion, UV-Vis spectra measurements were performed on a UV-1800 spectrophotometer (Shimadzu, Kyoto, Japan). Transmission Electron Microscopy (TEM) was performed using a field emission transmission electron microscope (JFM-2100F; JEOL, Tokyo, Japan). Images were acquired in the TEM mode using a 200 kV acceleration voltage. Samples were prepared by placing a droplet of the diluted GO dispersion directly onto the TEM grid.

### Preparation of urea resin/GO and test for formaldehyde emission

Table 3 summarises the mixing conditions of the UF resin and GO. A predetermined amount of GO as a formaldehyde scavenger, 0.04 g of ammonium chloride as a curing agent, and 4.00 g of liquid urea resin (including volatile content) were mixed for 1 min at 465 × *g* using a planetary centrifugal mixer (AR-100; Thinky, Tokyo, Japan). According to the manufacturer, the recommended amount of curing agent is 10 wt% against to the liquid UF resin.

**Table 3:** Compounding conditions of the UF/scavenger^a^

GO content^b^ [wt%]	GO [mg]	pH^c^ [—]
0	—	5.73
0.10	2.0	5.66
0.15	3.0	5.64
0.20	4.0	5.61
0.40	8.1	5.52
1.0	20.2	5.29
1.9	40.4	4.73

a Added amount of liquid urea resin (including volatile content) and curing agent were 4.00 g and 0.04 g, respectively.

b Weight ratio of the solid GO scavenger to the total weight of the solid content of UF resin, solid GO, and solid curing agent.

c pH measurement was conducted after adding the curing agent.

For comparison, as-received graphite was also used as a scavenger. The addition of graphite with 0.20–1.9 wt% to the total weight of the solid UF resin, solid graphite, and solid curing agent.

After curing the UF/scavenger for 1 h in an oven at 105 °C, the formaldehyde emission was measured by the desiccator method.^60^ It is noted that the curing reaction was carried out using an oven in this study. According to our previous study,^58^ this simple evaluation method for measuring a formaldehyde emission from UF resin was in good agreement with the emission from plywood. Specifically, the absorbance was measured at 412 nm using a UV-Vis spectrophotometer (UV-1800; Shimadzu, Japan). Emission tests were repeated six times for the control UF resin and three times for the UF/scavenger composite resins.

With 0.20, and 1.9 wt% GO content, the pH of UF/GO was regulated. Citric acid or calcium hydroxide, GO, UF resin, and a curing agent were combined for 1 min at 465 × *g* using a planetary centrifuge mill. After mixing, the mixture was cured for 1 h in an oven at 105 °C. Then the formaldehyde emission was evaluated as mentioned above.

### Gel time of urea resin

In order to investigate gelling property of UF resin, we measure the gel time of UF resin with regulating pH. A predetermined amount of citric acid, and 20.00 g of UF resin were mixed using an agitator. 0.20 g of curing agent was added to the mixture and time measurement was started. After the mixture was stirred vigorously for 5 min, the apparent viscosity at 100 rpm was monitored using a viscometer (DV-1 Prime RV, Eko instruments, Japan; upper limit of the viscosity at 100 rpm is 500 mPa s). The gel time was defined as a time beyond 500 mPa s in this study. The temperature was controlled at 50 °C during the viscosity measurement.

## Conclusions

Although DFT studies have noted that GO has an excellent adsorption capacity for formaldehyde, GO adsorption on formaldehyde-resin has yet to be experimentally investigated. This is the first experiment to demonstrate that GO effectively prevents formaldehyde emission from UF resin. Consistent with previous DFT calculations, GO is a suitable adsorbent material for inhibiting formaldehyde emission from UF resin. The UF resin containing 0.20 wt% GO exhibits the lowest formaldehyde emission of 0.22 ± 0.03 mg L^−1^, which was an 81.5% reduction compared to that of the control UF resin. The emission increases at GO contents below 0.20 wt% due to the lack of the adsorption site as well as above 0.20 wt% of the GO content. The pH of the UF/GO resin drops to acidic conditions, suggesting that formaldehyde is more predominantly diffused into the atmosphere than adsorption on the GO surface.

## Author contributions

Kazuki Saito: writing – original draft, formal analysis, investigation. Yasushi Hirabayashi: methodology, writing – review & editing. Shinya Yamanaka: project administration, supervision, funding acquisition, writing – review & editing.

## Conflicts of interest

There are no conflicts to declare.

## Supplementary Material
